# Risk factors for healthcare-associated candidemia in adults hospitalized with SARS-CoV-2 infection

**DOI:** 10.1017/ash.2024.25

**Published:** 2024-05-02

**Authors:** Zachary M. Most, John J. Hanna, Alexander P. Radunsky, Lauren N. Cooper, Christoph U. Lehmann, Trish M. Perl, Richard J. Medford

**Affiliations:** 1 Division of Infectious Disease, Department of Pediatrics, University of Texas Southwestern Medical Center, Dallas, TX, USA; 2 Division of Infectious Disease and Geographic Medicine, Department of Internal Medicine, University of Texas Southwestern Medical Center, Dallas, TX, USA; 3 Clinical Informatics Center, University of Texas Southwestern Medical Center, Dallas, TX, USA; 4 Peter O’Donnell School of Public Health, University of Texas Southwestern Medical Center, Dallas, TX, USA; 5 Lyda Hill Department of Bioinformatics, University of Texas Southwestern Medical Center, Dallas, TX, USA; 6 Division of Infectious Diseases, Department of Internal Medicine, Brody School of Medicine, Greenville, NC, USA; 7 Information Services, ECU Health, Greenville, NC, USA

## Abstract

Analyzing data from a national deidentified electronic health record-based data set using a matched case–control study design, we found that antibiotic use and severity of illness were independent risk factors for healthcare-associated candidemia in adult patients hospitalized with SARS-CoV-2 infection. Interleukin-6 inhibitor and corticosteroid use were not independent risk factors.

## Background

Patients hospitalized with severe acute respiratory syndrome coronavirus 2 (SARS-CoV-2) infection, the virus that causes COVID-19, are at increased risk for secondary infections with *Candida* spp., which can be severe and life-threatening.^1–4^ Prior studies identified risk factors for healthcare-associated candidiasis in patients hospitalized with COVID-19, including age, length of intensive care unit (ICU) stay, comorbidities, central venous catheters, mechanical ventilation, extracorporeal membrane oxygenation (ECMO), antibiotic exposure, and *Candida* colonization.^5–7^ It remains unclear if there are any risk factors for candidiasis that are unique to patients with COVID-19, including use of immunosuppressive medications such as corticosteroids and interleukin-6 (IL-6) inhibitors.

## Methods

We designed a nested matched case–control study using the Optum^®^ deidentified COVID-19 electronic health record (EHR) data set. The Optum^®^ COVID-19 data is a deidentified, national, low-latency pipeline that leverages data from inpatient and ambulatory EHRs, practice management systems, and other health information technology systems. The database captures over 4 million adults and children tested for COVID-19 from over 700 hospitals and 7,000 clinics in the United States. Due to the use of this expert-certified deidentified database, this study did not meet the definition of human subjects research and did not require institutional review board approval.

We identified cases and controls hospitalized between February 10, 2020, and June 26, 2021, who had a hospital admission within 14 days after testing positive on a polymerase chain reaction (PCR) or an antigen test for SARS-CoV-2. Cases had at least 1 blood culture growing any *Candida* spp. between 2 days and 30 days following hospital admission. *Candida* spp. included yeast with recent nomenclature changes to other genera.^8^ Controls had at least 1 blood culture growing another organism between 2 days and 30 days following hospital admission and no blood cultures growing *Candida* spp.

We matched controls to cases with a ratio of 4:1 on region as defined in the Optum^®^ COVID-19 data and on one of 4 periods of equal duration. We matched to ensure the balance between the groups on unmeasured location-time varying differences in COVID-19 illness severity and management. The index date was the date of the first blood culture growing an organism.

We compared various demographic, comorbidity, and medical intervention characteristics (see Supplemental Methods for definition of variables), using the Mann–Whitney test for quantitative variables and conditional logistic regression for categorical variables. We determined independent risk factors using multivariate conditional logistic regression with backward stepwise selection and used a two-sided *P* value of .05 for statistical significance. We assessed the interaction between period and IL-6 inhibitor and corticosteroid use using likelihood ratio tests. We conducted all analyses using Stata v16.1 (StataCorp, College Station, Texas, USA).

## Results

We identified 5,601 positive blood culture results (among 3,264 unique individuals) with SARS-CoV-2-associated hospitalizations in these data. After excluding individuals with positive cultures drawn before hospital day 3 and consolidating duplicates into the first positive culture, we identified 149 cases with *Candida* spp. (Table 1) growing in blood and 2,354 controls. Once we matched and excluded those with missing data, we analyzed 144 cases and 460 controls.

**Table 1.** *Candida* species observed (*n* = 149)

*Candida* species	No.	%
*C. albicans*	85	57.1
*C. glabrata*	30	20.1
*C. parapsilosis*	14	9.4
*C. tropicalis*	11	7.4
*C. dubliniensis*	4	2.7
*C. krusei*	2	1.3
*C. guilliermondii*	1	0.7
*C. kefyr*	1	0.7
*C. lusitaniae*	1	0.7
Total	149	100.0

Cases and controls had similar age (mean age 62.5 ± 16.0 years for cases and 63.6 ± 13.6 years for controls; *P* = .84), sex, and race/ethnicity (Table 2). Time from hospital admission to positive blood culture was similar for cases and controls (median, 13 vs 12 days; *P* = .11). In univariate analysis, antibiotic use, central venous catheter (CVC) use, and COVID-19 illness severity were associated with candidemia. IL-6 inhibitor use was more common in cases than controls, but the association did not reach statistical significance. Janus-associated kinase (JAK) inhibitor use was rare in this population (Table 2).

**Table 2.** Characteristics and comparisons of cases with candidemia and controls without

Variable	Category	Cases, *n*=144	Controls, *n*=460	OR (95% CI)	*P*	aOR (95% CI)	*P*
Age (years), no. (%)	18–49	27 (18.8)	62 (13.5)	1.00 (ref)	.37		
	50–64	47 (32.6)	169 (36.7)	0.67 (0.38–1.18)			
	65+	70 (48.6)	229 (49.8)	0.71 (0.41–1.21)			
Sex, no. (%)	Female	49 (34.0)	167 (36.3)	1.00 (ref)	.60		
	Male	95 (66.0)	293 (63.7)	1.11 (0.75–1.64)			
Race and ethnicity, no. (%)	NH Caucasian	68 (47.2)	211 (45.9)	1.00 (ref)	.12		
	NH African American	37 (25.7)	98 (21.3)	1.22 (0.76–1.96)			
	Hispanic	16 (11.1)	81 (17.6)	0.58 (0.30–1.09)			
	Asian^b^	23 (16.0)	17 (3.7)	1.85 (0.75–4.47)			
	NH Other/Unknown		53 (11.5)	0.88 (0.44–1.79)			
Region, no. (%)	Northeast	47 (32.6)	165 (35.9)		n/a		
	Midwest	50 (34.7)	156 (33.9)				
	South or West^b^ or Other/Unknown^b^	47 (32.7)	139 (30.2)				
Time Period, no. (%)	February 10, 2020–June 14, 2020	54 (37.5)	191 (41.5)		n/a		
	June 15, 2020–October, 17 2020	17 (11.8)	43 (9.4)				
	October, 18 2020–February 20 2021	50 (34.7)	145 (31.5)				
	February 21 202–June 26 2021	23 (16.0)	81 (17.6)				
Obesity, no. (%)	Underweight^b^	80 (55.6)	213 (46.3)	0.79 (0.21–2.93)	.14		
	Normoweight			1.00 (ref)			
	Obesity class 1 or 2	51 (35.4)	178 (38.7)	0.71 (0.47–1.09)			
	Obesity class 3	13 (9.0)	69 (15.0)	0.50 (0.26–0.98)			
Smoking (*n*=546), no. (%)	Current smoker	20 (15.8)	49 (11.7)		.38		
	Not current smoker	107 (84.3)	370 (88.3)				
CCI score	Median (IQR)	2 (5–8)	2 (5–8)	0.97 (0.92–1.02)	.28		
Hemi/paraplegia, no. (%)	Yes	23 (16.0)	47 (10.2)	1.63 (0.96–2.77)	.08		
	No	121 (84.0)	413 (89.8)	1.00 (ref)			
Hemodialysis, no. (%)	Yes	20 (13.9)	47 (10.2)	1.39 (0.76–2.54)	.29		
	No	124 (86.1)	413 (89.8)	1.00 (ref)			
HIV, no. (%)	Yes^b^	≤ 10	11 (2.4)	0.60 (0.13–2.70)	.47		
	No	> 134	449 (97.6)	1.00 (ref)			
TPN, no. (%)	Yes^b^	≤ 10	≤ 10	2.45 (0.14–42.6)	.54		
	No	> 134	> 450	1.00 (ref)			
Antibiotics, no. (%)	Yes	120 (83.3)	288 (62.6)	3.34 (2.00–5.57)	**< .001**	3.39 (1.99–5.76)	**< .001**
	No	24 (16.7)	172 (37.4)	1.00 (ref)		1.00 (ref)	
IL-6 inhibitor, no. (%)	Yes	27 (18.8)	79 (17.2)	1.27 (0.74–2.17)	.38		
	No	117 (81.3)	381 (82.8)	1.00 (ref)			
JAK inhibitor	Yes^b^	≤ 10	≤ 10	0.81 (0.08–8.52)	.86		
	No	> 134	> 450	1.00 (ref)			
Steroid^a^no. (%)	Yes	114 (79.2)	332 (72.2)	1.46 (0.92–2.32)	.10		
	No	30 (20.8)	128 (27.8)	1.00 (ref)			
ICU (prior), no. (%)	Yes	65 (45.1)	172 (37.4)	1.34 (0.90–2.01)	.15		
	No	79 (54.9)	288 (62.6)	1.00 (ref)			
Severity (prior), no. (%)	No ICU or vent	20 (13.9)	151 (32.8)	1.00 (ref)	**< .001**	1.00 (ref)	**< .001**
	ICU and no vent or ECMO	10 (6.9)	33 (7.2)	2.14 (0.89–5.20)		2.33 (0.94–5.77)	
	Vent or ECMO	114 (79.2)	276 (60.0)	3.93 (2.23–6.95)		3.98 (2.22–7.17)	
Central line (prior), no. (%)	Yes	54 (37.5)	122 (26.5)	1.85 (1.21–2.83)	**.004**		
	No	90 (62.5)	338 (73.5)	1.00 (ref)			

Unadjusted and adjusted odds ratios (OR) were calculated using conditional logistic regression models. Bolded values were statisticaly significant.

a Steroid includes receipt of ≥1 dose of dexamethasone, prednisone, prednisolone, or methylprednisolone prior to the culture date.

b Some cells were merged or hidden to ensure values >10 in all categories.

Smoking status was not considered in the univariate or multivariate model due to a large amount of missing data.

IL-6 (interleukin-6) inhibitors included tocilizumab or sarilumab.

JAK (Janus-associated kinase) inhibitor included baricitinib.

ICU, intensive care unit; vent, mechanical ventilation; ECMO, extracorporeal membrane oxygenation.

In multivariate analysis, antibiotic use prior to the index date and COVID-19 disease severity were independently associated with developing candidemia. There was no evidence of interaction with period when IL-6 inhibitor use and steroid use were individually added back to the model (*P* = .17 and *P* = .71, respectively).

## Discussion

In this matched case–control study, we identified antibiotic use and illness severity as independent risk factors for candidemia among adults hospitalized with SARS-CoV-2 infection. These findings are consistent with previously reported risk factors for candidemia. Importantly, our study did not demonstrate that either IL-6 inhibitor or steroid use was independently associated with candidemia.

The secondary impact of these immunomodulatory agents used to treat severe COVID-19 has been controversial, and results from prior studies of patients hospitalized with COVID-19 have been mixed. A study that combined candidiasis with invasive mold infections demonstrated that high-dose steroids increased the risk of fungal superinfections.^9^ Two ICU cohort studies with similar populations reached opposite conclusions regarding the association between steroids and candidemia.^5,6^ Furthermore, tocilizumab use did increase the risk for secondary fungal infections in patients with COVID-19 in a recent meta-analysis that did not distinguish between *Candida* and mold infections.^10^ Overall, our study provides further supporting evidence that IL-6 inhibitor and corticosteroid use in patients hospitalized with SARS-CoV-2 infection do not increase the risk of candidemia.

Our findings mirrored most other studies and identified antibiotic use as a risk factor for candidiasis. Although we identified illness severity as an independent risk factor, it was highly collinear with ICU stay and ECMO use. CVC use and ICU stay were not independent risk factors for candidemia once we adjusted for illness severity. However, including severe COVID-19 in the regression model may have modulated or masked their effects. It thus remains unclear how much hospital units, medical interventions, or patient-specific factors in those with severe COVID-19 contributed to secondary *Candida* bloodstream infections.

A strength of our study was the case sample size. Although we only included 144 cases, we defined these cases conservatively, and this study remains one of the largest studies to date of patients hospitalized with SARS-CoV-2 infection with secondary candidemia. Additionally, the use of test-negative controls with another pathogen identified from blood reduced selection bias by ensuring that we drew cases and controls from a similar acutely ill population, reduced the effect of false-negative blood cultures leading to misclassification of cases, and helped control for confounding by indication.

This study has several limitations. We assessed the effect of various cofactors, when possible, but we were limited in scope due to the data available in the deidentified data set. These data could be inaccurate, misclassified, or incomplete, especially for those variables defined using procedure codes. Additionally, there is a possibility of residual confounding due to data that may have not been captured. We only examined cofactors documented before the date of the positive blood culture, although it is possible the *Candida* infection could have started prior to the index time in some patients, which in theory could be a cause of, and not a result of, increased severity of illness. We did not have data on whether SARS-CoV-2 infection was the indication for hospital admission or was identified incidentally, so the results may or may not generalize to patients admitted for COVID-19.

In conclusion, the severity of illness and the use of antibiotics increased the risk of candidemia in adults hospitalized with SARS-CoV-2 infection. Further studies are needed to further define both the positive and negative effects of immunomodulating agents used to treat severe COVID-19 infections.

## Supporting information

Most et al. supplementary material Most et al. supplementary material

## Supplementary material

For supplementary material accompanying this paper visit http://doi.org/10.1017/ash.2024.25. click here to view supplementary material
