# Supplementary material for: Risk factors for healthcare-associated candidemia in adults hospitalized with SARS-CoV-2 infection
Source: Antimicrob Steward Healthc Epidemiol. 2024 May 2;4(1):e69. doi: 10.1017/ash.2024.25 (PMC11077599; doi:10.1017/ash.2024.25)
Supplement: Most et al. supplementary material [file S2732494X24000251sup001.docx]

Supplement to: Risk Factors for Healthcare-associated Candidemia in Adults Hospitalized with SARS-CoV-2 Infection

Supplemental Methods:

Further specification on selection of the study population:

- The database did not have reliable discharge dates so infections within 30 days of a SARS-CoV-2 associated hospital admission were used as a proxy for infections occurring during the index hospitalization.
- We only used blood culture results because cultures from non-sterile collection sites do not definitively establish infection and positive cultures from other sterile sites were rare in the data.
- Controls with positive blood cultures were used because they 1) served as proven test negative controls (definitively did not have candidemia) and 2) likely had similar indications for obtaining blood cultures.
- Individuals with separate blood cultures positive for *Candida* spp. and another organism during the same hospitalization were included as cases, and the index date was the date of the first positive blood culture for *Candida* spp.
- For variables with only a small amount of missing data, individuals were excluded from the analysis if they had missing data.
- Backward stepwise selection: in the multivariable model we initially included all variables with *P*<0.10 in univariable regression, then removed all variables with *P*>0.10 sequentially from the model.

We evaluated the following potential risk factors:

- demographics (age, sex, race/ethnicity),
- obesity,
- smoking status
- medical comorbidities
- total parental nutrition (TPN) administration
- medical interventions (hemodialysis, antibiotic use, IL-6 inhibitor use, Janus kinase (JAK) inhibitor use, and corticosteroid use)
- intensive care unit (ICU) stay
- presence of a central venous catheter (CVC)
- severity of COVID-19.

Age was grouped into three categories as labeled in the Optum^®^ COVID-19 data (18-49 years, 50-64 years, 65+ years). We combined race and ethnicity into groups as labeled in Optum^®^ COVID-19 data (Hispanic, Non-Hispanic African American, Non-Hispanic Caucasian, Asian, Other). We classified body mass index (BMI) into four categories (underweight, normoweight, obesity class 1 or 2, obesity class 3). Smoking status was listed as current smoker or not current smoker. Individual comorbidities and the Charlson comorbidity index (CCI) were captured. TPN, hemodialysis catheter, CVC, antibiotic use, IL- 6 inhibitor use, JAK inhibitor use, corticosteroid use, and ICU stay of one or more days were all coded dichotomously and extracted from procedure code dates, medication administration, or care area data. These variables were considered between admission to (and including) the hospital day prior to the index date. Corticosteroids were defined as at least one administration of: dexamethasone, prednisone, prednisolone, or methylprednisolone of any dose. Illness severity was an ordinal variable with three levels: no ICU stay, ICU stay and no mechanical ventilation or ECMO, and mechanical ventilation or ECMO.
